# Supplementary material for: Reconstruction of fallout deposition from U.S. atmospheric nuclear tests conducted in New Mexico and Nevada
Source: Sci Rep. 2026 Aug 24;16:23550. doi: 10.1038/s41598-026-62206-x (PMC13503652; doi:10.1038/s41598-026-62206-x)
Supplement: Supplementary file 1 [file 41598_2026_62206_MOESM2_ESM.pdf]

Supplementary information for

Reconstruction of fallout deposition from U.S. atmospheric nuclear tests conducted in New Mexico and Nevada

Sébastien Philippe[1,2,*], Susan Alzner[3], Gilbert P. Compo[4,5], Mason Grimshaw[6], Megan Smith[3]

[1] Department of Nuclear Engineering and Engineering Physics, University of Wisconsin, Madison, Wisconsin

[2] Program on Science and Global Security, Princeton University, Princeton, New Jersey

[3] shift7, Washington, District of Columbia

[4] Cooperative Institute for Research in Environmental Sciences, University of Colorado Boulder, Boulder, Colorado

[5] Physical Sciences Laboratory, NOAA, Boulder, Colorado

[6] Ode Partners, Rapid City, South Dakota

* Correspondence to: sphilippe@wisc.edu

This file includes one supplementary table and six supplementary figures.

Supplementary Table 1. List of 101 atmospheric nuclear tests conducted in New Mexico and Nevada with key reconstruction parameters. Tests marked (*) were not included in this study (see main text).

Test	U.S. #	Operation	Name	Date	Type	Time (GMT)	Latitude	Longitude	Height [m AGL]	Yield [kt]	Cloud top [m AGL]	Cloud bottom [m AGL]
1	1	MANHATTAN	Trinity	7/16/45	Tower	11:29	33.6773	-106.4754	30.5	24.8	9259	1822
2	7	RANGER	Able	1/27/51	Airdrop	13:45	36.8051	-115.9502	323.1	1	4225	2841
3	8	RANGER	Baker	1/28/51	Airdrop	13:52	36.8051	-115.9502	329.2	8	9711	6531
4	9	RANGER	Easy	2/1/51	Airdrop	13:47	36.8051	-115.9502	329.2	1	2853	1919
5	10	RANGER	Baker-2	2/2/51	Airdrop	13:49	36.8051	-115.9502	335.3	8	7577	5096
6	11	RANGER	Fox	2/6/51	Airdrop	13:47	36.8051	-115.9502	437.4	22	12149	7273
7	16	BUSTER	Able	10/22/51	Tower	14:00	37.0833	-116.0247	30.5	0.1	1168	771
8	17	BUSTER	Baker	10/28/51	Airdrop	15:20	37.0850	-116.0211	340.8	3.5	8384	5732
9	18	BUSTER	Charlie	10/30/51	Airdrop	15:00	37.0850	-116.0213	345	14	11219	6952
10	19	BUSTER	Dog	11/1/51	Airdrop	15:30	37.0846	-116.0209	431.9	21	12743	8171
11	20	BUSTER	Easy	11/5/51	Airdrop	16:30	37.0919	-116.0253	400.5	31	13953	9381
12	21	JANGLE	Sugar	11/19/51	Surface	17:00	37.1315	-116.0394	1.2	1.2	3287	2068
13	23	TUMBLER-SNAPPER	Able	4/1/52	Airdrop	17:00	36.7980	-115.9364	241.7	1	4000	2690
14	24	TUMBLER-SNAPPER	Baker	4/15/52	Airdrop	17:30	37.0841	-116.0203	338	1	3507	1770
15	25	TUMBLER-SNAPPER	Charlie	4/22/52	Airdrop	17:30	37.0844	-116.0211	1050.7	31	11524	8171
16	26	TUMBLER-SNAPPER	Dog	5/1/52	Airdrop	16:30	37.0841	-116.0211	317	19	12133	7256
17	27	TUMBLER-SNAPPER	Easy	5/7/52	Tower	12:15	37.0530	-116.1035	91.4	12	9044	5711
18	28	TUMBLER-SNAPPER	Fox	5/25/52	Tower	12:00	37.0955	-116.1036	91.4	11	11184	7062
19	29	TUMBLER-SNAPPER	George	6/1/52	Tower	11:55	37.0955	-116.1036	91.4	15	10050	6347
20	30	TUMBLER-SNAPPER	How	6/5/52	Tower	11:55	37.1384	-116.1183	91.4	14	11372	7181
21	33	UPSHOT-KNOTHOLE	Annie	3/17/53	Tower	13:20	37.0955	-116.1036	91.4	16	11270	7307
22	34	UPSHOT-KNOTHOLE	Nancy	3/24/53	Tower	13:10	37.0955	-116.1036	91.4	24	11336	6611
23	35	UPSHOT-KNOTHOLE	Ruth	3/31/53	Tower	13:00	37.0853	-116.0221	93	0.2	2926	2042
24	36	UPSHOT-KNOTHOLE	Dixie	4/6/53	Airdrop	15:30	37.0846	-116.0207	1835.5	11	12489	8832
25	37	UPSHOT-KNOTHOLE	Ray	4/11/53	Tower	12:45	37.0989	-116.0933	30.5	0.2	2674	1120
26	38	UPSHOT-KNOTHOLE	Badger	4/18/53	Tower	12:35	37.1384	-116.1183	91.4	23	9604	5642
27	39	UPSHOT-KNOTHOLE	Simon	4/25/53	Tower	12:30	37.0530	-116.1035	91.4	43	12119	8157

28	40	UPSHOT-KNOTHOLE	Encore	5/8/53	Airdrop	15:30	36.7979	-115.9298	738.5	27	11864	7597
29	41	UPSHOT-KNOTHOLE	Harry	5/19/53	Tower	12:05	37.0403	-116.0262	91.4	32	11733	7161
30	42	UPSHOT-KNOTHOLE	Grable	5/25/53	Airburst	15:30	36.7979	-115.9298	159.7	15	9730	6073
31	43	UPSHOT-KNOTHOLE	Climax	6/4/53	Airdrop	11:15	37.0875	-116.0192	406.6	61	11788	9441
32	50	TEAPOT	Wasp	2/18/55	Airdrop	20:00	37.0865	-116.0227	232.3	1	5275	3141
33	51	TEAPOT	Moth	2/22/55	Tower	13:45	37.0955	-116.1036	91.4	2	6149	3619
34	52	TEAPOT	Tesla	3/1/55	Tower	13:30	37.1254	-116.0484	91.4	7	7918	4352
35	53	TEAPOT	Turk	3/7/55	Tower	13:20	37.1384	-116.1183	152.4	43	12256	9330
36	54	TEAPOT	Hornet	3/12/55	Tower	13:20	37.0403	-116.0262	91.4	4	10056	7252
37	55	TEAPOT	Bee	3/22/55	Tower	13:05	37.0948	-116.0246	152.4	8	10807	7698
38	57	TEAPOT	Apple-1	3/29/55	Tower	12:55	37.0955	-116.1036	152.4	14	8440	5575
39	58	TEAPOT	Wasp Prime	3/29/55	Airdrop	18:00	37.0865	-116.0227	225.2	3	8475	5700
40	59	TEAPOT	HA	4/6/55	Airdrop	18:00	37.0287	-116.0587	9931.1	3	15533	10446
41	60	TEAPOT	Post	4/9/55	Tower	12:30	37.1221	-116.0353	91.4	2	3433	2366
42	61	TEAPOT	MET	4/15/55	Tower	19:15	36.7979	-115.9298	121.9	22	11345	8755
43	62	TEAPOT	Apple-2	5/5/55	Tower	12:10	37.0530	-116.1035	152.4	29	14254	9225
44	63	TEAPOT	Zucchini	5/15/55	Tower	12:00	37.0948	-116.0246	152.4	28	10898	6387
45	64*	Project 56	No. 1	11/1/55	Surface	22:10	36.9844	-115.9616	0.9	0	n/a	n/a
46	65*	Project 56	No. 2	11/3/55	Surface	21:15	36.9801	-115.9595	0.9	0	n/a	n/a
47	66*	Project 56	No. 3	11/5/55	Surface	19:55	36.9757	-115.9575	0.9	$3.6\ 10^{-8}$	n/a	n/a
48	67	Project 56	No. 4	1/18/56	Surface	21:30	36.9713	-115.9554	0.9	0.001	914	610
49	87	PLUMBBOB	Boltzmann	5/28/57	Tower	11:55	37.0948	-116.0245	152.4	12	8768	5720
50	88	PLUMBBOB	Franklin	6/2/57	Tower	11:55	37.0955	-116.1036	91.4	0.14	3863	3040
51	89	PLUMBBOB	Lassen	6/5/57	Balloon	11:45	37.1347	-116.0416	152.4	0.0005	722	486
52	90	PLUMBBOB	Wilson	6/18/57	Balloon	11:45	37.1347	-116.0416	152.4	10	9379	6331
53	91	PLUMBBOB	Priscilla	6/24/57	Balloon	13:30	36.7979	-115.9298	213.4	37	12169	6378
54	92*	PLUMBBOB	Coulomb-A	7/1/57	Surface	17:30	37.0412	-116.0269	0	0	n/a	n/a
55	93	PLUMBBOB	Hood	7/5/57	Balloon	11:40	37.1347	-116.0416	457.2	74	13341	9379
56	94	PLUMBBOB	Diablo	7/15/57	Tower	11:30	37.1502	-116.1095	152.4	17	8392	4734
57	95	PLUMBBOB	John	7/19/57	Rocket	14:00	37.1604	-116.0538	4520.2	2	12104	7644
58	96	PLUMBBOB	Kepler	7/24/57	Tower	11:50	37.0955	-116.1036	152.4	10	7221	4783
59	97	PLUMBBOB	Owens	7/25/57	Balloon	13:30	37.1347	-116.0416	152.4	9.7	9383	4811
60	99	PLUMBBOB	Stokes	8/7/57	Balloon	12:25	37.0865	-116.0248	457.2	19	10002	6954
61	101	PLUMBBOB	Shasta	8/18/57	Tower	12:00	37.1279	-116.1073	152.4	17	8417	3540
62	102	PLUMBBOB	Doppler	8/23/57	Balloon	12:00	37.0865	-116.0248	457.2	11	10293	5721
63	104	PLUMBBOB	Franklin Prime	8/30/57	Balloon	12:40	37.0865	-116.0248	228.6	4.7	8478	5125

64	105	PLUMBBOB	Smoky	8/31/57	Tower	12:30	37.1872	-116.0687	213.4	44	10217	6452
65	106	PLUMBBOB	Galileo	9/2/57	Tower	12:40	37.0530	-116.1035	152.4	11	9982	3886
66	107	PLUMBBOB	Wheeler	9/6/57	Balloon	12:45	37.1347	-116.0416	152.4	0.197	3892	2978
67	108	PLUMBBOB	Coulomb-B	9/6/57	Surface	20:05	37.0429	-116.0271	0.9	0.3	4257	2688
68	109	PLUMBBOB	Laplace	9/8/57	Balloon	13:00	37.0865	-116.0248	228.6	1	4820	2991
69	110	PLUMBBOB	Fizeau	9/14/57	Tower	16:45	37.0334	-116.0322	152.4	11	10964	7001
70	111	PLUMBBOB	Newton	9/16/57	Balloon	12:50	37.0865	-116.0248	457.2	12	8478	4515
71	113	PLUMBBOB	Whitney	9/23/57	Tower	12:30	37.1384	-116.1183	152.4	19	7777	4119
72	114	PLUMBBOB	Charleston	9/28/57	Balloon	13:00	37.1347	-116.0416	457.2	12	8469	4811
73	115	PLUMBBOB	Morgan	10/7/57	Balloon	13:00	37.1347	-116.0416	152.4	8	10908	6640
74	117	PROJECT58	Coulomb-C	12/9/57	Surface	20:00	37.0482	-116.0252	0	0.5	2728	1723
75	160	HARDTACKII	Eddy	9/19/58	Balloon	14:00	37.0865	-116.0248	152.4	0.083	2077	1010
76	165	HARDTACKII	Mora	9/29/58	Balloon	14:05	37.0865	-116.0248	457.2	2	4363	1772
77	166	HARDTACKII	Hidalgo	10/5/58	Balloon	14:10	37.0865	-116.0248	114.9	0.077	2382	1163
78	169	HARDTACKII	Quay	10/10/58	Tower	14:30	37.0948	-116.0245	30.5	0.079	1754	992
79	170	HARDTACKII	Lea	10/13/58	Balloon	13:20	37.0865	-116.0248	457.2	1.4	3906	2382
80	172	HARDTACKII	Hamilton	10/15/58	Tower	16:00	36.8023	-115.9331	15.2	0.0012	890	433
81	174	HARDTACKII	Dona Ana	10/16/58	Balloon	14:20	37.0865	-116.0248	137.2	0.037	2077	705
82	175	HARDTACKII	Vesta	10/17/58	Surface	23:00	37.1226	-116.0354	0	0.024	1760	1111
83	176	HARDTACKII	Rio Arriba	10/18/58	Tower	14:25	37.0409	-116.0268	22.3	0.09	2893	2131
84	178	HARDTACKII	Socorro	10/22/58	Balloon	13:30	37.0865	-116.0248	442	6	6649	4820
85	179	HARDTACKII	Wrangell	10/22/58	Balloon	16:50	36.7979	-115.9298	457.2	0.115	2110	1196
86	180*	HARDTACKII	Oberon	7/1/57	Tower	20:30	37.1828	-116.0693	7.6	0	n/a	n/a
87	181	HARDTACKII	Rushmore	10/22/58	Balloon	23:40	37.1347	-116.0416	152.4	0.188	457	307
88	182	HARDTACKII	Catron	10/24/58	Tower	15:00	37.0428	-116.0278	22.3	0.021	1364	297
89	183	HARDTACKII	Juno	10/24/58	Surface	16:01	37.1233	-116.0387	0	0.0017	393	248
90	184	HARDTACKII	Ceres	10/26/58	Tower	4:00	37.1813	-116.0695	7.6	0.0007	479	303
91	185	HARDTACKII	Sanford	10/26/58	Balloon	10:20	36.7979	-115.9298	457.2	4.9	6987	2872
92	186	HARDTACKII	De Baca	10/26/58	Balloon	16:00	37.0865	-116.0248	457.2	2.2	4058	1772
93	187	HARDTACKII	Chaves (Chavez)	10/27/58	Tower	14:30	37.0446	-116.0307	15.8	0.0006	754	476
94	189*	HARDTACKII	Mazama	10/30/58	Tower	11:20	37.1265	-116.0419	15.2	0	n/a	n/a
95	190	HARDTACKII	Humboldt	10/29/58	Tower	14:45	37.0476	-116.0254	7.6	0.0078	1058	601
96	191	HARDTACKII	Santa Fe	10/30/58	Balloon	3:00	37.0865	-116.0248	457.2	1.3	4211	2687
97	192*	HARDTACKII	Ganymede	10/30/58	Surface	11:00	37.1265	-116.0479	0	0	n/a	n/a
98	194	HARDTACKII	Titania	10/30/58	Tower	20:34	37.1772	-116.0701	7.6	0.0002	487	307
99	265	SUNBEAM	Little Feller II	7/7/62	Surface	17:00	37.1191	-116.3037	0.9	0.022	1790	1130
100	271	SUNBEAM	Small Boy	7/14/62	Tower	18:30	36.8025	-115.9259	3	1.65	4853	3065
101	272	SUNBEAM	Little Feller I	7/17/62	Surface	17:00	37.1096	-116.3182	0.9	0.018	1770	1118

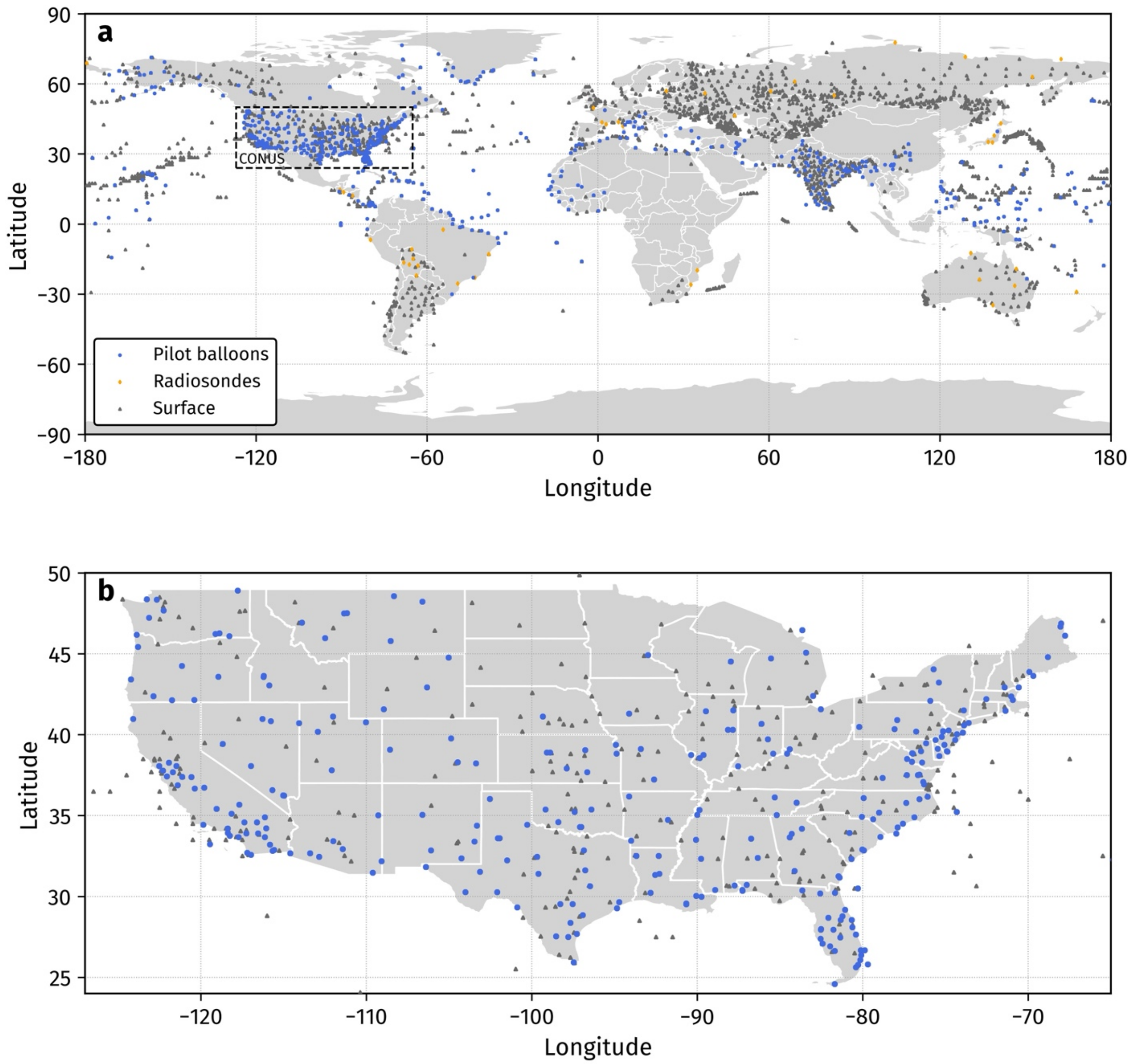

Supplementary Figure 1. ERA5 surface and upper air (pilot balloons and radiosondes) observations for July 16, 1945. (**a**) World-wide observations. (**b**) Details for the region of interest in this study.

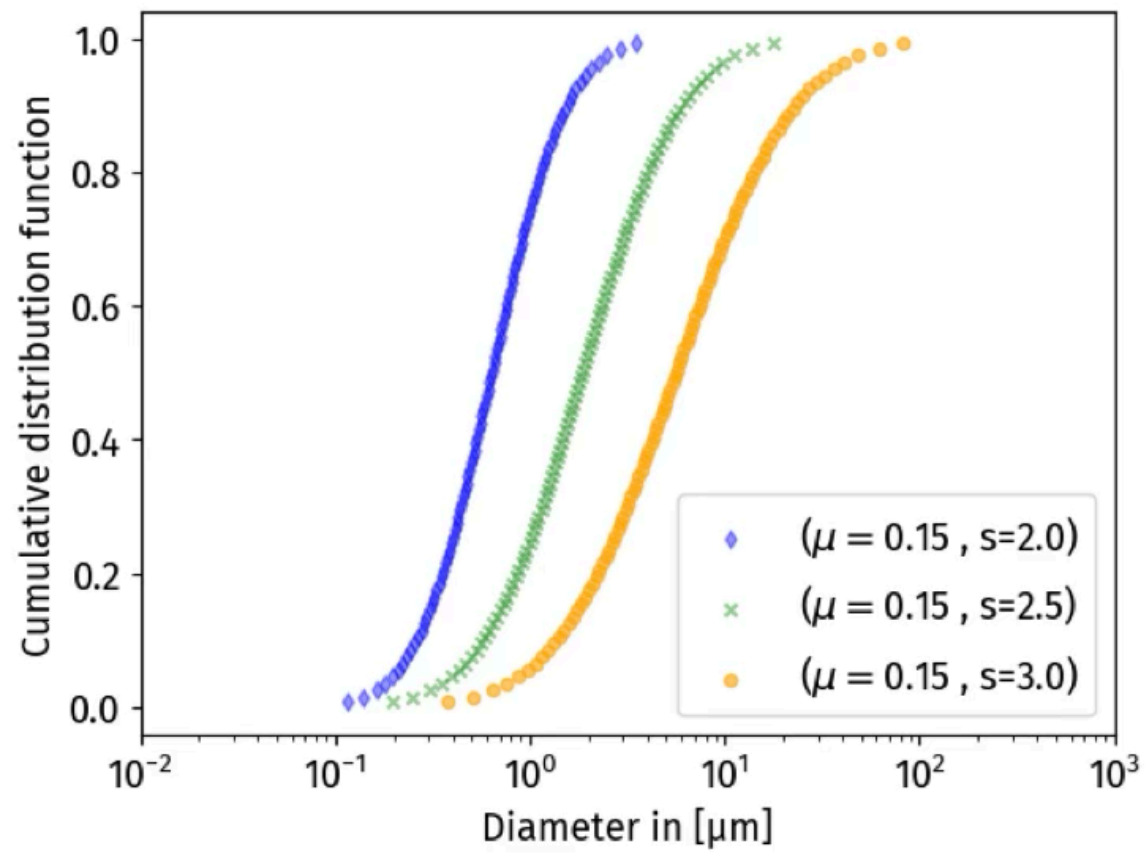

Supplementary Figure 2. Cumulative distribution function (CDF) of particle diameters for three log-normal size distributions used in airburst reconstructions. All distributions share the same median particle diameter (μ = 0.15 μm) but differ in geometric standard deviation (s = 2.0, 2.5, and 3.0). Increasing the standard deviation parameter (s) results in a broader distribution and a greater fraction of larger particles. These distributions were tested to benchmark model output against National Cancer Institute iodine-131 data (see Figure 1).

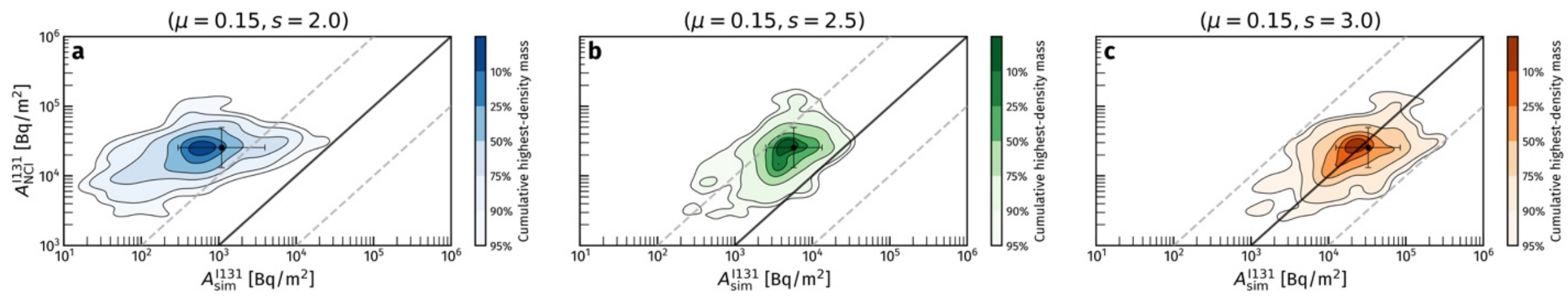

Supplementary Figure 3. Comparison between HYSPLIT-reconstructed and National Cancer Institute (NCI) county-level iodine-131 deposition estimates for airburst tests, using three different log-normal particle size distributions. Each panel shows the joint distribution of reconstructed (x-axis) versus NCI-reported (y-axis) iodine-131 deposition densities (in Bq m^{-2}), evaluated with a two-dimensional kernel density estimate (KDE) in log-space. Colored regions denote *highest-density regions* (HDRs) containing the densest 10%, 25%, 50%, 75%, 90%, and 95% of the probability mass. Results are shown for a fixed median particle diameter ($\mu = 0.15$ µm) and three geometric standard deviations: $s = 2.0$ (**a**), $s = 2.5$ (**b**), and $s = 3.0$ (**c**). The solid black line indicates perfect 1:1 agreement, while dashed gray lines show ±1 order-of-magnitude deviations. Increasing geometric spread systematically improves agreement with NCI observational estimates, with the $s = 3.0$ distribution producing the closest match across counties. Points with error bars represent the mean reconstructed and observed deposition values with $\pm 1\sigma$ log-space dispersion.

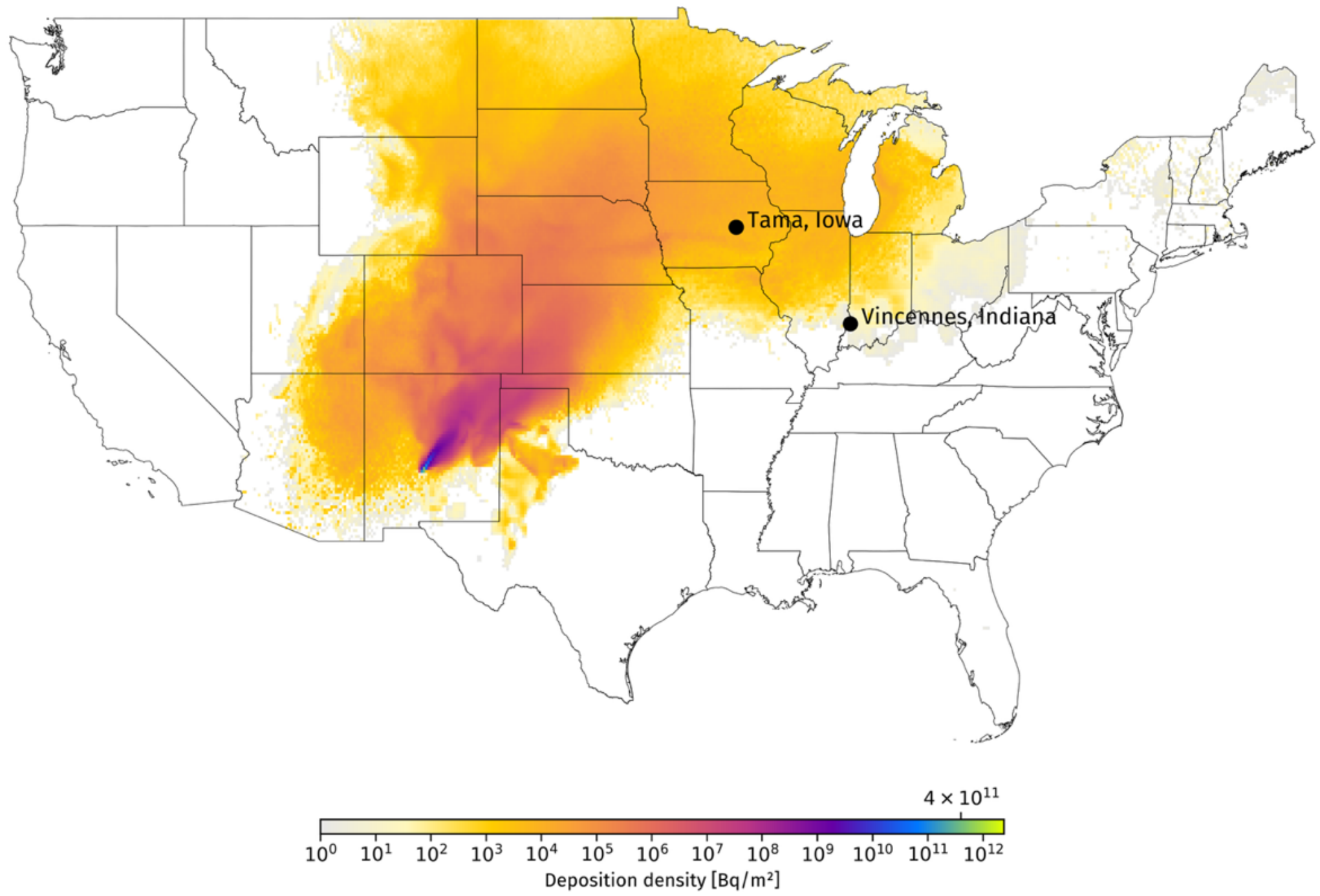

Supplementary Figure 4. Reconstructed cumulative deposition density from the Trinity test six days after detonation, showing long-range fallout consistent with 1945 photographic film contamination. In August 1945, the Eastman Kodak Company reported unexplained fogging on X-ray film packaged with strawboard manufactured at mills in Vincennes, Indiana, and Tama, Iowa. Radiochemical analysis of the contaminated board revealed beta-emitting isotopes with an approximately 30-day half-life, later attributed to fission products such as cerium-141. This was among the earliest documented instances of long-range fallout from Trinity. Our reconstruction of fission-product deposition (in Bq/m^2) from the Trinity test shows northeastward transport of radioactive material, including deposition over Indiana and Iowa as of July 22, 1945 when fallout begins to reach Vincennes, Indiana.

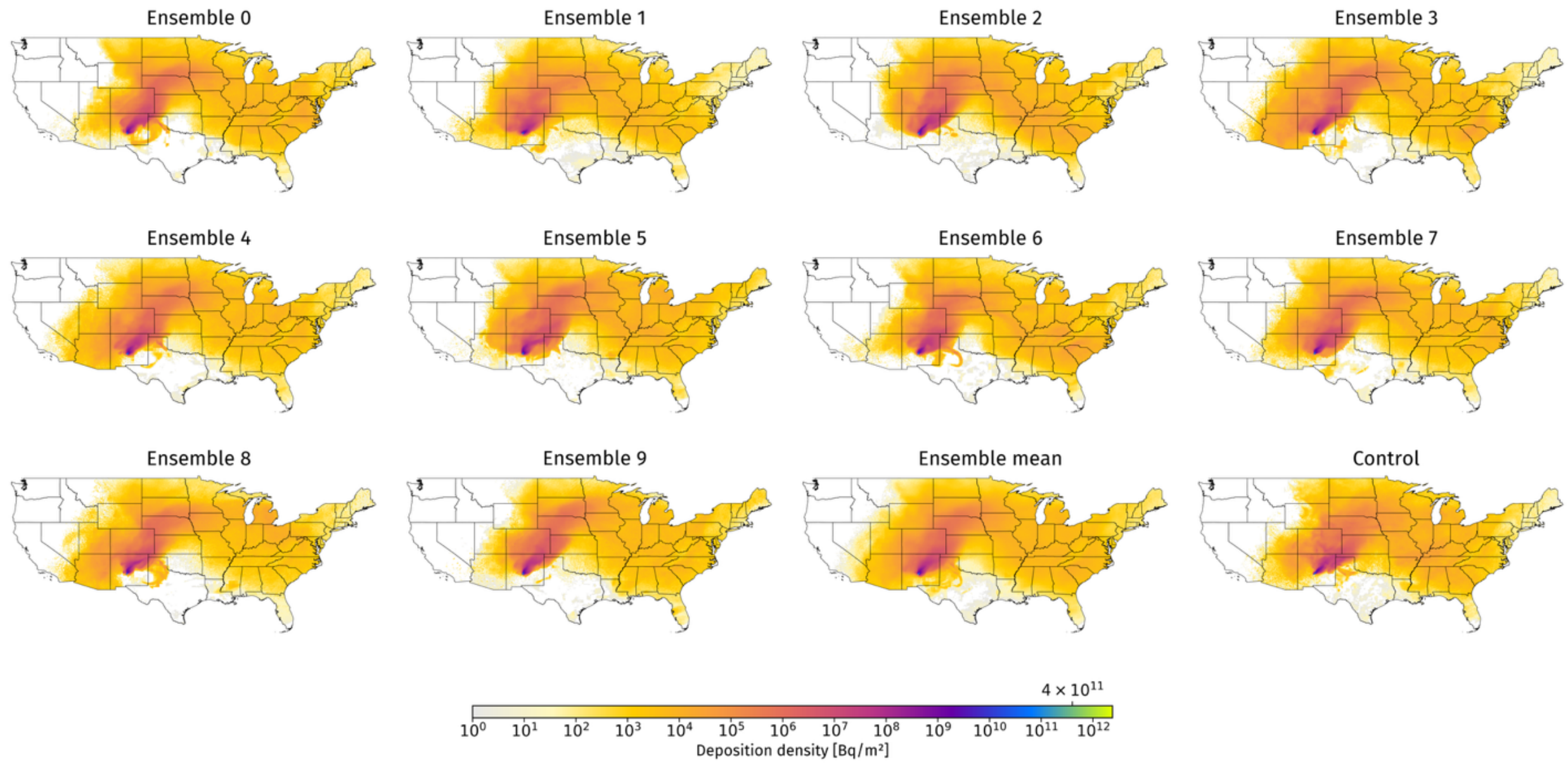

Supplementary Figure 5. Ten-day cumulative fission-product deposition from the Trinity test using each ERA5 ensemble member, the ensemble mean, and the reanalysis (control) data set. All reconstruction ensemble members use identical source terms, transport parameters, and spatial grids. While the spatial structure and intensity of fallout vary modestly between ensemble members, the overall geographic extent of deposition is consistent, with material reaching at least 45 of the United States in all members. These results highlight long-range transport features common to all realizations.

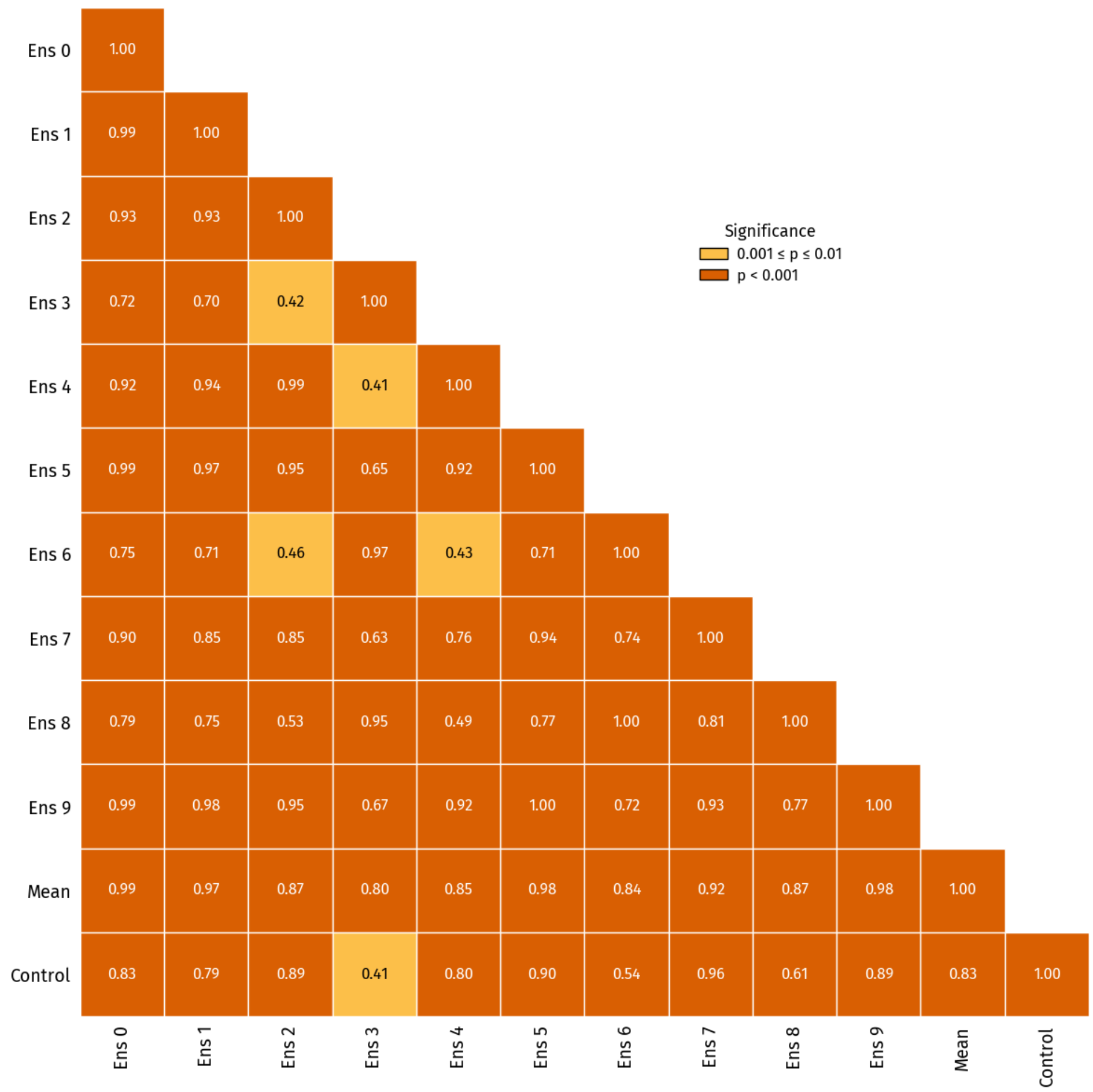

Supplementary Figure 6. Pairwise Pearson correlation matrix for Trinity fallout deposition across ERA5 ensemble members and the control. Matrix of Pearson correlation coefficients computed between the 10 ensemble members, the ensemble mean, and the control reconstruction for Trinity 10-day cumulative deposition fields. All values exceed the two-tailed significance threshold of 0.36 at $p = 0.01$, assuming 48 degrees of freedom (based on 5° × 5° independent blocks across the 20° × 60° reconstruction domain). Correlation values range from 0.41 to 0.99 between ensemble pairs, and from 0.41 to 0.96 between each ensemble member and the control, indicating strong spatial agreement in modeled deposition patterns across the ensemble spread.
